# The Effect of Zirconia Material and the Height of the Ceramic Coping on the Strength of Hybrid Ti-Ceramic Abutments

**DOI:** 10.3390/dj13070284

**Published:** 2025-06-23

**Authors:** Aikaterini Anastasaki, Pranit Bora, Stefanos Kourtis, Chin Chuan Fu

**Affiliations:** 1 Department of Prosthodontics, College of Dental Medicine, Columbia University, New York, NY 10032, USA; aa5679@cumc.columbia.edu; 2 Division of Clinic Essentials and Simulation, School of Dentistry, University of Detroit Mercy, Detroit, MI 48221, USA; borapv@udmercy.edu; 3 Department of Prosthodontics, Dental School, National and Kapodistrian University of Athens, 11527 Athens, Greece; 4 Department of Restorative Sciences, School of Dentistry, University of Alabama at Birmingham, Birmingham, AL 35294, USA; ccfu@uab.edu

**Keywords:** zirconia, titanium base abutment, crown height

## Abstract

The existing scientific literature lacks comprehensive information regarding the influence of zirconia crown height on debonding and fracture of the ceramic restorations on titanium base abutments. Additionally, there is a lack of comparative studies evaluating different types of zirconia as restorative options for screw-retained restorations. **Purpose**: The purpose of this study was to assess the fracture strength and the failure modes of the zirconia crown/titanium abutment complex by investigating the impact of increasing the height of zirconia crown and comparing different types of zirconia (3 mol% yttria-stabilized zirconia and translucent 5 mol% yttria-stabilized zirconia). **Materials and Methods**: Six groups of 10 specimens in each group were fabricated. Three groups of specimens (groups # 1, 2, and 3) were fabricated from 3Y zirconia in corresponding heights of 8, 10, and 12 mm. Three more groups (groups # 4, 5, and 6) were fabricated from 5Y zirconia in the same heights (8, 10 and 12 mm). All copings were bonded to 4 mm high titanium base abutments using dual-polymerization resin cement. The specimens underwent load cycling of 100,000 cycles with a force of 100 N. Subsequently, the specimens were loaded to compression until fracture and the failure mode was visually evaluated. **Results**: Statistically significant differences in fracture strength were noted among all tested groups. **Conclusions**: 3Y zirconia showed increased strength compared to 5Y in all heights. Ceramic copings with lower height showed increased strength compared to higher copings in both tested zirconia materials.

## 1. Introduction

Prosthetic restoration is a factor of major importance for the clinical life of implants. The choice of materials for abutments, the intermediate structures between the implant and crown, has evolved with options like titanium, chrome-cobalt, zirconia, and gold [1]. Titanium abutments gained prominence due to compatibility with CAD/CAM technology, enabling faster working times and improved adaptation [2]. Their retrievability, ability to be cemented extraorally, and seamless integration into the CAD/CAM workflow make titanium abutments advantageous [3].

Zirconia (ZrO_2_) has been used widely in recent years for several applications in dentistry as it combines strength, biocompatibility, and esthetic performance [4]. Recently, there have been significant improvements in restorative biomaterials including dental zirconia, and a wide variety of zirconia has been produced for prosthetic restorations in dentistry [5,6].

The abutment fracture rate of zirconia was reported as 1.08% to 17.86% depending on age, gender, tooth position, abutment systems, implant systems, and implant–abutment connections. Zirconia abutments in the posterior areas were more susceptible to fractures, compared to the anterior areas [7]. The risk of abutment fracture may also be affected by the thickness of the material and the position and angulation of the implant [8,9]. To decrease the risk of an abutment fracture, the wall thickness of zirconia abutments must be maintained above 0.5 mm during manufacturing, while the wall thickness of the titanium abutment should not be thinner than 0.5 mm [7].

The fracture resistance of hybrid abutments (titanium base supporting monolithic zirconia restorations) is affected by the angulation as shown in an in vitro study [10]. The strength was shown at a maximum for 15° angulations while at an angulation of 25°, it was similar to the straight abutments. The fracture resistance of titanium and zirconia abutments was investigated in an extended systematic review based on 17 papers [11]. The highest strength was noted for titanium abutments while titanium–zirconia two-piece abutments showed similar strength. One-piece zirconia abutments had the lowest value and were recommended by the authors for anterior restorations.

The use of ceramic materials for implant abutments has shown very good results regarding the response of the crestal bone and the peri-implant soft tissues [8,12]. In an extended systematic review (based on systematic reviews) the titanium abutments had a better mechanical resistance compared to ceramic abutments [9]. On the other hand, the esthetic performance of ceramic materials, especially in cases of thin peri-implant tissue, favors their clinical use [13]. In a systematic review on the survival of implants, a lower survival rate was found for implants with zirconia abutments compared to titanium abutments, despite the favorable effect of the ceramic material on peri-implant health [14].

Abutments with a titanium base and a ceramic crown or a ceramic coping serving as an individualized basis for a crown (also mentioned as hybrid or two-piece abutments) are widely acceptable in current clinical practice as they combine the fit of the industrial fabricated base (that will fit in the interior of the implant) with the esthetic performance of ceramic materials [15,16]. In an in vitro study comparing zirconia, PEEK, and lithium disilicate on hybrid abutments, zirconia showed the highest strength while the other materials can cautiously be used up to the premolar region [12].

However, the success of the titanium base concept relies on reliable cementation between the titanium base and the crown. Various surface treatments have been explored, with studies evaluating their effects on adhesion strength [17,18]. The type of cement and cleaning procedure used also plays a critical role, with studies highlighting the importance of selecting appropriate luting cement [19,20].

The height of the titanium base abutment is another factor influencing stability, with conflicting findings in the literature [21,22,23]. While some studies advocate higher abutments for increased retention [21], others emphasize the significance of adhesive resin-based cements regardless of abutment height [23].

The influence of abutment height on the marginal bone loss around two-piece abutments was investigated in a systematic review of clinical trials [24]. The authors concluded that longer abutments may prevent marginal bone loss compared to shorter abutments. Similar results had been reported in a previous systematic review [25].

On the other hand, bone resorption, especially in the posterior regions, may make necessary the use of short implants with crowns of increased length and a non-favorable implant-to-crown relation. The clinical results on the technical complications of short implants—and probably longer crowns—have so far been controversial [26].

Previous systematic reviews reported no difference in the rate of technical complications [27,28] while others indicated increased complications at specific follow-up recalls in short implants [29]. The increased crown-to-implant ratio, commonly observed in short implants, may be a risk factor for prosthetic complications [28]. A systematic review focused on the complications related to the crown-to-implant ratio considered an unfavorable ratio as a potential factor for technical problems [30]. On the other hand, a previously published [31] and a recent systematic review [32] on the crown–implant ratio could not associate an increased rate of technical complications in crowns with increased length.

The abovementioned influence of the abutment height and the selection of the proper ceramic material on the strength of the implant–abutment complex prompted the present investigation.

### Aim

The purpose of this study was to assess the failure modes of the zirconia crown/titanium abutment complex by investigating the impact of increasing the height of the zirconia crown and comparing different types of zirconia (3 mol% yttria-stabilized zirconia and translucent 5 mol% yttria-stabilized zirconia).

The null hypothesis was that no difference among abutments with different heights would be observed. Similarly, there would be no difference between the two tested types of zirconia.

## 2. Materials and Methods

For the experimental procedure, 6 groups of 10 specimens in each group were fabricated. Three groups of specimens (groups # 1, 2, and 3) were fabricated from 3Y zirconia in corresponding heights of 8, 10, and 12 mm. Three more groups (4, 5, and 6) were fabricated from 5Y zirconia in the same heights (8, 10, and 12 mm. The implant analogs were PYIA Internal 3.5 mm analogs (Biohorizons Inc., Birmingham AL USA), and the titanium bases were PYHYB 3.5 mm Hybrid Abutment Bases (Biohorizons Inc., USA), with their corresponding fixing screws being PXMUAS (Biohorizons Inc., USA). The ceramic copings were fabricated from Zirlux (Henry Schein Co., Melville, NY, USA). For the 3Y zirconia, the Zirlux 16+ material was used and for the 5Y copings, the Zirlux Anterior Multi was used. The ceramic copings were cemented to the titanium abutments with Panavia V5 (Kuraray Co., Tokyo, Japan). The groups of specimens are presented in Table 1.

### 2.1. Fabrication of Zirconia Copings

**Figure 1:**
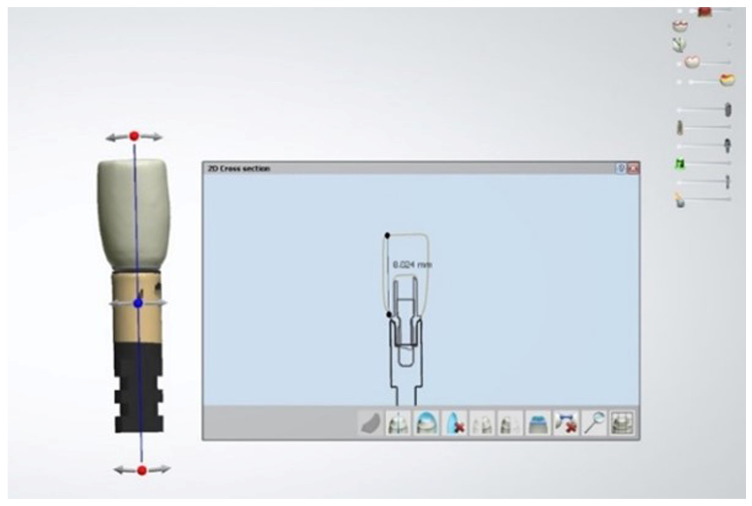
CAD coping design.

**Figure 2:**
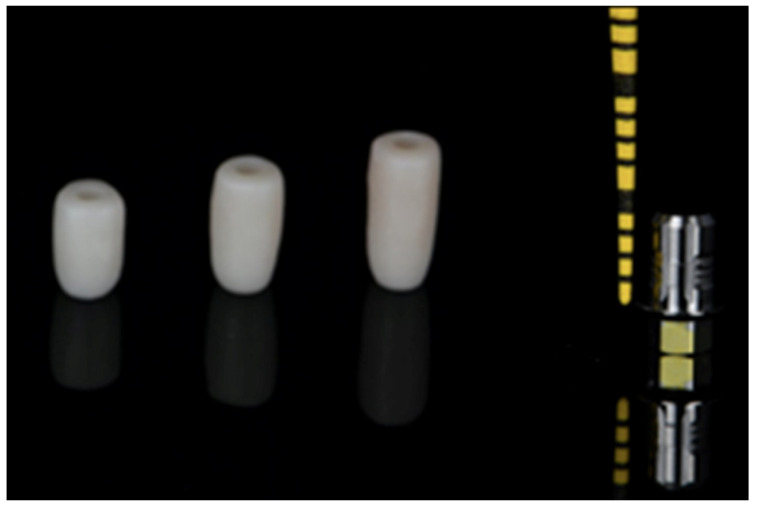
Zirconia copings with 8, 10, and 12 mm height and the titanium base (3.5 mm diameter and 4 mm height).

The copings were designed using CAD software (Millbox 2020 CNC Software, CIM System, Balsamo, Italy), milled in a Roland DWX-52d dry mill device (Roland DGA Co., Irvine, CA, USA), and sintered in a zirconia sintering furnace (Sintra Plus, Shenpaz Co., New York, NY, USA) to ensure structural integrity. After this step, the copings were carefully examined for any deformation or debris under magnification and were corrected as necessary. Subsequently, the copings were cleaned using a steamer machine (Steaman Cleaner Junior, Bar Instruments, Henri Scein Co., Melville, NY, USA) (Figure 1 and Figure 2).

**Figure 3:**
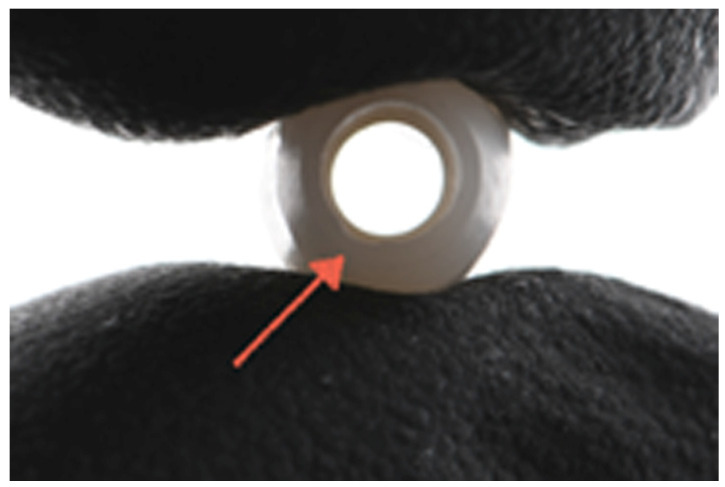
Orientation notch on coping.

To complete the assembly, copings of each height were tried in on commercially available titanium bases (3.5 mm titanium base, BioHorizons Inc., Birmingham, AL, USA). These titanium bases are cylindrical in shape and fabricated from Ti-6Al-4v Grade 5. The bases have a facial height of 4 mm and a palatal height of 2 mm. The different coping heights represented titanium-base/coping height ratios of 1:1, 1:1.5, and 1:2, corresponding to clinical crowns with low, medium, and increased height. The titanium base was securely screwed into an implant analog and torqued to 30 N/cm. The access hole of the base was filled with Teflon tape, and the path of insertion of the crown was verified using orientation notches on the intaglio surface of the crown (Figure 3). The intaglio surface of the zirconia copings was sandblasted using 50 μm alumina (Renfert Cobra sandblaster, Renfert Co., Hilzingen, Germany) at 2 bar pressure for 20 s. After sandblasting, a 10-MDP-containing primer (Clearfil Ceramic Primer Plus, Kuraray Co., Tokyo, Japan) was applied both to the internal surfaces of the copings and the titanium bases using a micro brush for 20 s. The surfaces were blown with dry air for 5 s and polymerized for 10 s. The zirconia copings were bonded to the titanium bases using a resin cement (Panavia V5, Kuraray Co., Tokyo, Japan).

During the seating process, all crowns were subjected to a constant force of 10 N, using a custom-made device that ensured consistent and controlled pressure. Excess cement was carefully removed using a micro brush, and the cement layer was light-polymerized for 20 s on each side of the crown using an LED curing light (Bluephase G2, Ivoclar-Vivadent Co., Vadouz, Liechtenstein) with an intensity of 1200 mW/cm^2^.

### 2.2. Fatigue Loading

**Figure 4:**
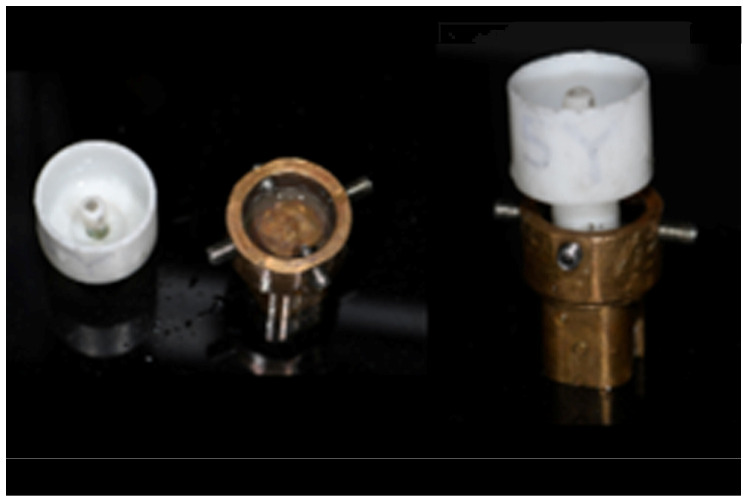
The 3D-printed mold for securing the assembly of the coping and the analog.

Following the completion of the bonding procedures, the zirconia copings, along with their respective titanium bases, were transferred and adapted to a custom-made 3D-printed mold specifically designed to securely hold the specimens during fatigue loading (Figure 4).

The specimens underwent fatigue loading in the vertical direction with 40 N force for 100,000 cycles at 1.7 Hz/s speed to simulate one year of intra-oral use, utilizing a custom fatigue device, often referred to as *Modified Alabama Wear Device*.

### 2.3. Compressive Loading Tests

**Figure 5:**
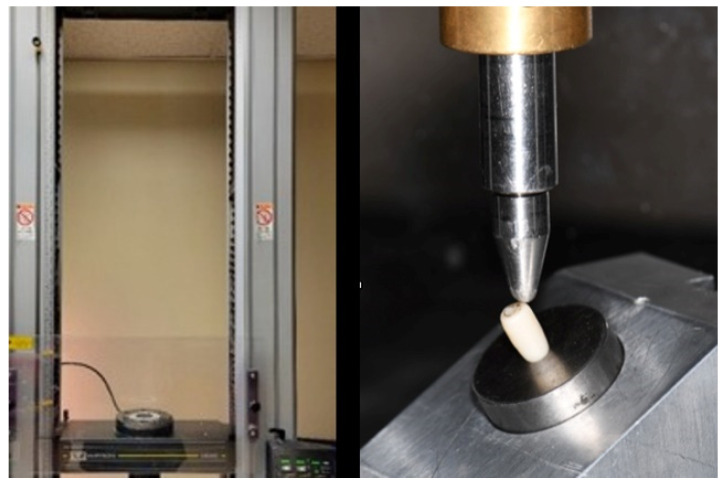
Compressive test.

Following fatigue loading, specimens were subjected to compressive tests up to fracture in a universal testing machine (Instron 5583, Instron Inc., Norwood, MA, USA). A custom fixture at a 30° angle inclination simulated occlusal force application (Figure 5). After loading, visual inspection and the assessment of failure modes were conducted. Load-to-failure values were recorded for comparative analysis among different groups.

## 3. Results

**Figure 6:**
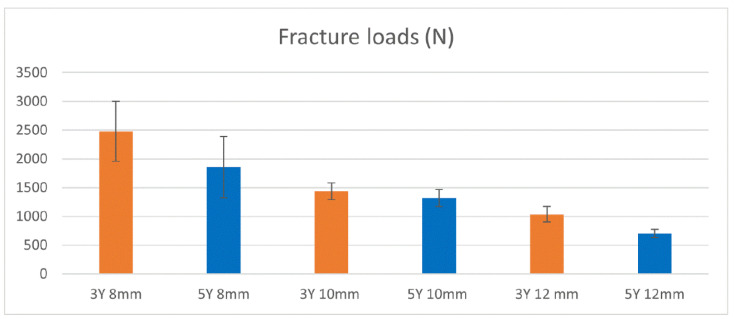
Mean ± standard deviation of failure strength (N) of ceramic copings.

The results of the ultimate failure strength for the different groups (mean and standard deviation) are presented in Figure 6. The values in the vertical axis indicate the fracture load, and the groups of specimens are shown in the horizontal axis.

The 2-way ANOVA analysis revealed a statistically significant main effect both for the parameter “material” l (*p* < 0.001) and the parameter “height” (*p* < 0.001), with a non-significant interaction between “material” and “height” (*p* = 0.052) (Table 2).

Additionally, the 3Y zirconia material demonstrated significantly higher failure force compared to the 5Ygroup for all tested heights. The study yielded significant findings, rejecting both null hypotheses and indicating that both crown height and zirconia material have substantial effects on the load to failure of zirconia crowns bonded on titanium abutments (Table 2).

For a more detailed investigation, a post hoc Tukey HSD analysis (Table 3) revealed distinct groups based on crown height.

The results of this study can be summarized as following: 3Y copings showed higher strength in all tested groups of different heights, suggesting that 3Y should be preferred for the fabrication of all ceramic restorations on Ti abutments. The height of the ceramic coping negatively affected their strength; copings with 8 mm height showed higher strength compared to the 10 mm copings, followed by the 12 mm group, in both tested ceramic materials. From a clinical point of view, ceramic crowns with increased height may be more susceptible to fracture compared to shorter crowns.

### Failure Modes

**Figure 7:**
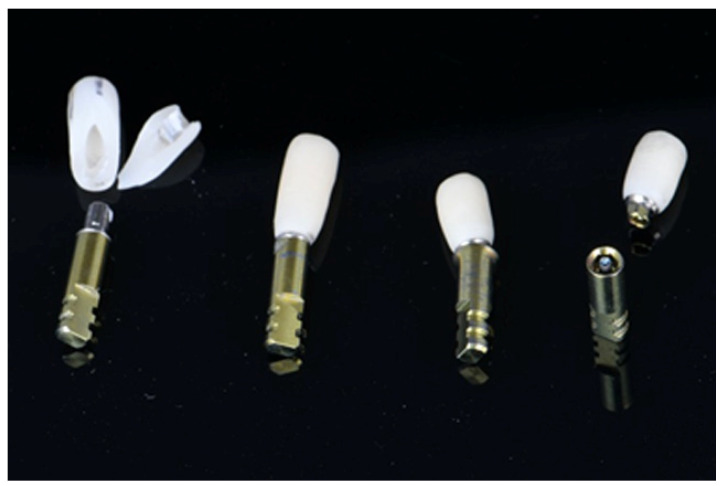
Failure modes of 3Y zirconia copings.

The failure modes varied between crown height and zirconia material. For 3Y zirconia crowns, the 8 mm height group displayed complete debonding, complete debonding with fracture, screw deformation, and screw fracture. The 10 mm group mainly experienced complete debonding, while the 12 mm group saw complete debonding with fracture and debonding with screw deformation (Figure 7).

**Figure 8:**
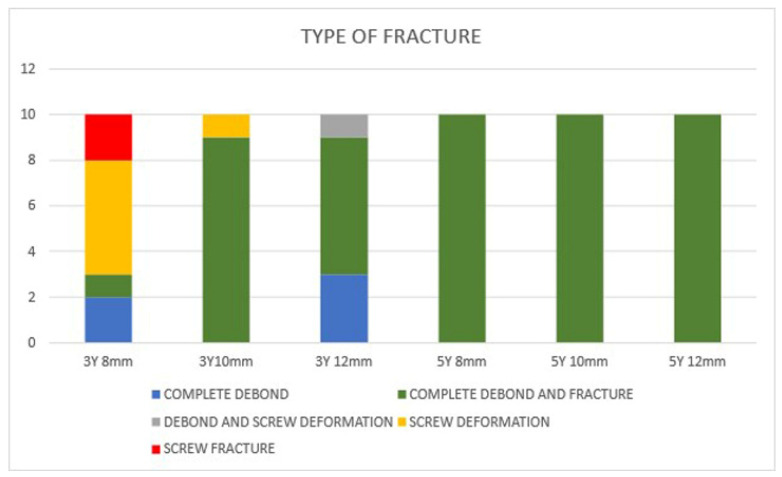
Types of fractures for all zirconia copings.

In the group of 5Y zirconia crowns, all height groups exhibited a combination of debonding and fracture, suggesting different mechanical responses to varying crown heights and zirconia materials (Figure 8). In the vertical axis, the number of groups are shown (a total of 10 specimens per group) and in the horizontal axis the different groups are shown. Different colors indicate the various fracture types in the corresponding number.

## 4. Discussion

The use of screw-retained restorations is currently expanding in clinical practice due to the advantages of retrievability and the lack of cements. Hybrid abutments combine the advantages of screw retention with individualized shape and fabrication using CAD/CAM technology. An important factor for the clinical performance of these abutments is the connection of the prefabricated titanium base to the ceramic coping. This connection is influenced by various parameters such as the surface treatment of the components [17,18], the type of cement [15,16], and the height of the titanium base or ceramic coping [21,22,23]. The connection of the ceramic copings to the titanium bases is based on the use of dual-polymerization cements with their corresponding bonding agent to achieve a stable bond between the two materials [15,16,17,18]. The combined treatment of sandblasting of the ceramic material and the use of MDP-10 has shown a positive effect on the bond strength of titanium and ceramic [17].

The results of this study showed different failure forces for the various heights of the copings. Similar differences were observed for the two tested zirconia materials. The rejection of the null hypotheses underscores the independent and substantial effects of both crown height and zirconia material, highlighting their pivotal roles in the strength and resilience of these restorations.

The findings of this study indicate that crown height plays a crucial role in the load to failure, as supported by the post hoc Tukey test revealing distinct groups based on crown height. The 8 mm height exhibited the highest failure resistance, followed by 10 mm and 12 mm. The clinical interpretation of the results is that shorter crowns are more resistant compared to crowns with increased height, as a lower leverage effect is created. These findings corroborate an earlier study [32], underlining the importance of crown height in implant restorations. On the contrary, crowns with increased height (as it is usually the case in bone resorption and short implants) showed reduced ultimate failure force.

The association between higher zirconia crowns and increased mechanical complications rates may be attributed to higher leverage forces, creating elevated stress concentrations at the zirconia–titanium interface as longer crowns exert higher forces to the implant through an elongated abutment. This renders the restoration more susceptible to technical problems, such as debonding, fractures, or screw loosening [33].

The influence of crown height on the strength of the implant–abutment complex was investigated in an in vitro study [34]. Ceramic crowns (lithium disilicate and zirconia-reinforced lithium disilicate) of different heights (7.5/10.5 and 13.5 mm) were refabricated on titanium abutments of standard height (5 mm). The implant–crown complex was loaded to fracture. The height of crowns negatively affected the strength of the complex in both tested materials, as longer crowns showed reduced values compared to shorter ones.

The findings of this study concerning the increased strength of shorter compared to longer copings can be associated with the negative effect of the increased crown-to-implant ratio on the survival of implants and the rate of technical complications [30]. A critical threshold value of 3.40 for the crown-to-implant ratio has been suggested to avoid implant failure and technical complications, such as in clinical situations with very short implants [35]. On the other hand, according to a systematic review on short implants, the crown-to-implant ratio did not affect implant survival or the rate of technical problems [36].

Regarding the height of Ti abutments, an in vitro study evaluated the influence of Ti base height on the strength of the abutment–ceramic coping complex [37]. Ti-base abutments of different heights (1, 3, and 5 mm) were attached to zirconia copings of a standard height of 13 mm and tested for deformation after thermocycling. The fracture strength in all groups was lower than the values recorded in the present study. The 1 mm Ti abutments showed statistically significant values compared to the 3 and 5 mm groups, which showed no difference. The type of zirconia that was used was not reported. Relating the results of this study to the findings of the present study, it can be assumed that the reduced support of the ceramic material from the titanium abutment may negatively influence the strength of the hybrid abutment.

Comparing the 3Y and 5Y zirconia groups, the present study revealed a significant difference in fracture resistance, as 3Y copings showed increased strength compared to the 5Y group. This finding holds substantial implications for clinical decision-making, suggesting that 3Y zirconia offers enhanced strength and durability, favoring its use for implant restorations exposed to significant occlusal forces and mechanical stresses. The influence of ceramic material on the strength of hybrid abutments has already been investigated, and titanium base abutments with zirconia coping have shown the potential for average occlusal forces [38]. These outcomes resonate with the existing literature on the mechanical properties of zirconia materials. Previous studies have also emphasized the importance of zirconia type selection in implant restorations [39,40]. The mechanical properties of the 3Y zirconia material, as highlighted in the present study, offer valuable information for clinicians in optimizing long-term success and durability.

The findings of the present study agree with a previous study where 3Y zirconia showed higher fracture resistance compared to 5Y, probably due to the cubic phase content because of the higher yttria content [41]. In another in vitro study [42], where untreated blanks of zirconia from various dental laboratories were tested for flexural strength, 3Y zirconia showed increased strength values (158 MPa) compared to 5Y zirconia (104 Mpa). The fracture of 3Y specimens showed intergranural fracture while the 5Y specimens exhibited transgranural fracture.

The addition of yttria (Y_2_O_3_) to zirconia stabilizes the tetragonal phase. Yttria-doping can reduce grain growth, stabilize the tetragonal phase, and substantially improve thermal stability. This doping of zirconia results in partially stabilized zirconia (PSZ) [43]. The yttria-stabilized dental zirconia is classified into different types based on the yttria content [4]. Zirconia with a lower yttria content (3Y-TZP, 3 mole % Y-TZP) has better mechanical properties and less translucency whereas 3Y-TZP (3 mole % Y-TZP) with an increased yttria content (6Y-TZP, 6 mole %Y-TZP) has more translucency but presents lower mechanical properties. Yttria content consisting of 3–8 mol% has tetragonal and cubic phases and is known as partially stabilized zirconia (PSZ). Yttria content consisting of approx. 3 mol% has tetragonal phases (toughened) about 100% and is known as a tetragonal zirconia polycrystal (TZP) [4].

Although the higher content of yttria increases translucency, a favorable characteristic for anterior restorations with limited extent, it reduces the mechanical properties. A decrease in fracture toughness has also been shown for 4Y-TZP zirconia compared to 3 mol% yttria, and the latter is considered as a favorable combination to balance aging and mechanical properties [44,45].

The fracture toughness of 5Y-TZP was almost 50% less compared to that of 3Y-TZP due to the cubic phase content because of the higher yttria content [41]. In a recent study [42], the flexural strength value of 3Y-TZP zirconia was significantly higher compared to 5Y-TZP.

On the other hand, zirconia is a ceramic material with increased strength but remains brittle. For this reason, hybrid abutments have recently gained increased acceptance from clinicians. Therefore, a proper selection of zirconia material should be performed for the crown depending on whether esthetics or strength is needed.

The effect of the height of a screw-retained zirconia implant crown on a Ti base abutment was investigated in an in vitro study [46]. The authors concluded that the crown height did not affect detorque values, and 14 mm crowns had similar torque loss to shorter crowns after cyclic loading. On the other hand, the survival time to failure of the 14 mm crown–implant complex was shorter, also resulting in fractures of screws and implants.

The effect of different heights of Ti base abutments and the type of cement on traction retention in Zr-based restorations was investigated in an in vitro study [23]. A Ti base abutment of 4 mm height demonstrated retention similar to the 2.5 mm abutments. On the other hand, the kind of cement affected the retention of the crowns, and resin-based cement showed the highest value.

The effect of Ti base height, type of resin cement, and yttria-stabilized tetragonal zirconia polycrystal (Y-TZP) (Zr) surface pretreatment and/or Ti base abutment on the traction retention of implant-supported crowns was also investigated in another in vitro study [47]. The results demonstrated a direct relationship between Ti base height, micromechanical and/or chemical pretreatment, and the kind of cementation.

The effect of abutment height on the strength of implant-supported single ceramic crowns has been shown in an in vitro study [48]. Titanium abutments of various heights were bonded to monolithic ceramic crowns and fractured after thermocycling. The authors concluded that the height of the titanium base was the predominant factor impacting fracture resistance, superseding the influence of other geometric features (ex anti-rotational features).

The clinical use of titanium abutments with ceramic crowns has been documented in a clinical trial with a low incidence of prosthetic complication but with a short-term follow-up of 1 year [49]. On the other hand, a systematic review [2] concluded that although hybrid abutments are an adequate choice for implant restorations, there is limited evidence to support their mechanical performance, and highlighted the need for further research. Similar results were also reported in other recent systematic reviews [50,51].

Furthermore, the examination of only two zirconia materials (3Y and 5Y) limits the generalization of the results. Future investigations should encompass a broader range of zirconia materials commonly used in clinical practice to provide a more comprehensive understanding of their mechanical performance and failure modes.

While load to failure remains a primary outcome measure, it would be beneficial for future studies to explore additional mechanical properties, such as fatigue resistance and fracture toughness. These would offer a more comprehensive assessment of zirconia crowns’ performance on titanium base abutments.

In conclusion, both null hypotheses were rejected. The present study underscores the critical role of crown height and zirconia material in determining the load to failure of implant crowns bonded on titanium abutments. The association between shorter crown heights and higher failure forces necessitates careful consideration in treatment planning, especially in scenarios involving significant bone loss and large restorative spaces.

The higher mechanical strength of the 3Y zirconia material compared to 5Y zirconia advocates for its consideration in screw-retained implant restorations. However, these findings should be validated through further research that explores additional influencing factors and evaluates various mechanical properties.

## 5. Conclusions

Within the limitations of this study, the following can be concluded:

- The height of the ceramic coping negatively affected the strength of the tested titanium base hybrid abutment. Short ceramic copings showed higher failure forces in both tested zirconia materials.
- The 3Y zirconia copings showed increased strength compared to 5Y zirconia in all height groups.

## Figures and Tables

**Table 1:** Groups of specimens.

3YZ	8 mm Group 1	10 mm Group 2	12 mm Group 3
5YZ	8 mm Group 4	10 mm Group 5	12 mm Group 6

**Table 2:** Two-way ANOVA for the parameters “material” and “height”.

Tests of Between-Subjects Effects					
Dependent Variable: Load					
Source	Type III Sum of Squares	df	Mean Square	F	Sig.
Corrected Model	19,677,166.995 ^a^	5	3,935,433.399	37.947	0.000
Intercept	129,883,362.548	1	129,883,362.548	1252.381	0.000
Height	17,107,829.702	2	8,553,914.851	82.480	0.000
Material	1,923,269.165	1	1,923,269.165	18.545	0.000
Height * Material	646,068.128	2	323,034.064	3.115	0.052
Error	5,600,294.524	54	103,709.158		
Total	155,160,824.066	60			
Corrected Total	25,277,461.518	59			

^a^. R Squared = 0.778 (Adjusted R Squared = 0.758).

**Table 3:** Post hoc Tukey HSD analysis.

Tukey B ^a,b^				
Height (mm)	N	Subset		
		1	2	3
12	20	869.6035		
10	20		1376.9680	
8	20			2167.3275
Means for groups in homogeneous subsets are displayed. ^a,b^ Based on observed means. The error term is mean square (error) = 103,709.158.				

^a^. Uses harmonic mean sample size = 20,000. ^b^. Alpha = 0.05.

## Data Availability

The raw data supporting the conclusions of this article will be made available by the authors on request.
